# Automatic Prosodic Analysis to Identify Mild Dementia

**DOI:** 10.1155/2015/916356

**Published:** 2015-10-19

**Authors:** Eduardo Gonzalez-Moreira, Diana Torres-Boza, Héctor Arturo Kairuz, Carlos Ferrer, Marlene Garcia-Zamora, Fernando Espinoza-Cuadros, Luis Alfonso Hernandez-Gómez

**Affiliations:** ^1^Center for Studies on Electronics and Information Technologies, Universidad Central “Marta Abreu” de Las Villas, 54830 Santa Clara, Cuba; ^2^Center for Elderly Adults No. 2, 54830 Santa Clara, Cuba; ^3^Signal, Systems and Radiocommunications Department, Universidad Politécnica de Madrid, 28040 Madrid, Spain

## Abstract

This paper describes an exploratory technique to identify mild dementia by assessing the degree of speech deficits. A total of twenty participants were used for this experiment, ten patients with a diagnosis of mild dementia and ten participants like healthy control. The audio session for each subject was recorded following a methodology developed for the present study. Prosodic features in patients with mild dementia and healthy elderly controls were measured using automatic prosodic analysis on a reading task. A novel method was carried out to gather twelve prosodic features over speech samples. The best classification rate achieved was of 85% accuracy using four prosodic features. The results attained show that the proposed computational speech analysis offers a viable alternative for automatic identification of dementia features in elderly adults.

## 1. Introduction

Dementia is a disorder characterized by an impairment of intellectual and communicative functioning, with high impact among elderly people. Usually this disorder leads to dependency of the patient on their families or caregivers due to the impossibility to carry out their daily tasks. A general agreement of the experts in the field revealed that the number of patients with dementia is increasing around the world due to a progressive aging society [1].

With this fact in mind the early detection of the dementia syndrome becomes an important goal to slow down the development of cognitive deterioration, allowing either the use of alternative nonpharmacological therapies or short periods of pharmacological treatments. Current methods of dementia screening in older adults involve structured interviews. Questionnaire tests such as the Mini-Mental State Examination (MMSE) [2], Clinical Dementia Rating (CDR) [3], or Memory Impairment Screen (MIS) [4] are commonly used. These methods typically rely on prolonged interviews with the patient and a family member. Therefore, an automated method for screening of dementia is highly desirable.

Due to the fact that one of the most significant areas affected by dementia is language, many researches have been oriented towards speech analysis, showing that language impairment is strongly related to cognitive impairment. Even more, the first clues start to appear some years before patient is clinically diagnosed [5, 6].

In this paper we propose a framework that applies speech signal analysis to identify mild dementia (MD). In contrast to previous works in automating the evaluation of cognitive impairment through speech analysis that relied on manual transcripts of audio recording, our system uses a novel method for automatically detecting syllable nuclei in order to measure prosodic features without the need for a transcription. In the next sections we present an overview of our data, followed by the description of the feature extraction procedure we propose and the classification technique used to determine whether the subject has mild dementia or not.

## 2. Methods

### 2.1. Experimental Subjects

Within the framework of this exploratory work, speech recordings were conducted at the Center of Elderly Adult #2 in Santa Clara, Cuba. A total of twenty subjects were selected for this pilot experiment from a group of candidates. Our sample comprises participant older than sixty years old with a diagnosis of mild dementia and healthy subjects. Other inclusion criteria were basic reading skills and no significant visual impairments. All the work was performed strictly following the ethical consideration of the center and the participants were notified about the research and their agreement obtained.  Table 1 shows demographic data, with no significant differences between groups in terms of gender, age, or years of education.

### 2.2. Recording Tools and Procedures

Also trying to make the process of speech recording the less annoying as possible for a daily clinical practice, a specific tool was developed. It consisted of the use of a standard laptop equipped with two headworn condenser C520L AKG microphones for capturing both clinician and patient voices. Each microphone was connected to a different channel (left or right) of an M-audio ADC device connected to the laptop through a USB port. This configuration provides some acoustic separation, despite no complete isolation, of patient and clinician voices, thus making it easier for their processing.

**Figure 1:**
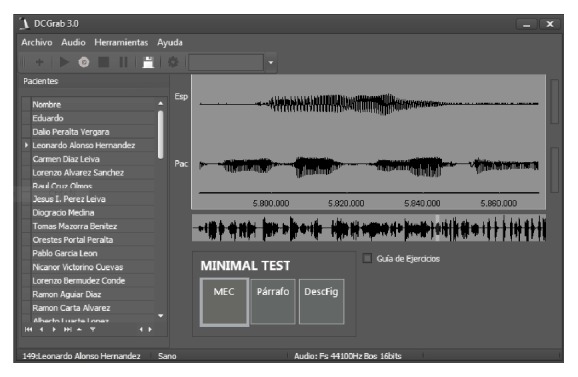
DCGrab v3.0 software.

A specific software DCGrab v3.0 was created by the authors to allow an easy recording of the audio signals during each one of the parts defined in our recording protocol. The speech sound was recorded in stereo format with 16 bits of resolution and 44.1 KHz of sampling rate. The DCGrab v3.0 software also allows storing clinical data and demographic information for each patient (see Figure 1).

### 2.3. Protocol and Speaking Styles

Two major conflicting criteria were considered for the design of the recording protocol in our database. On one side, it should represent the minimum possible burden on the busy schedules of daily clinical practices, while on the other side it should collect the richest variety of speaking styles which can result in a notable increase in testing time.

Consequently we decided to design a protocol consisting of two sequential parts. During the first part we recorded the speech productions from both clinician and patient during structured interviews commonly used in clinical assessment procedures. More specifically we considered the* Mini Examen Cognoscitivo* (MEC) which is the Spanish version of the Mini-Mental State Examination (MMSE) [2]. The MEC evaluation is our gold standard to classify each participant and evaluate the automatic method proposed in this work.

The second part of the interview consists of asking each enrolled subject to read a Spanish version of the paragraph “The Grandfather Passage” [7]:  *¿Tú quieres saber todo acerca de mi abuelo? Pues bien, él tiene casi 93 años de edad y aún conserva su mente tan brillante como siempre. Él usa un viejo saco negro al que generalmente le faltan algunos botones. Su barba larga y colgante produce en quienes lo observan un profundo sentimiento de máximo respeto. Cuando el habla, su voz es un poco rajada y tiembla ligeramente. Dos veces al día toca un pequeño órgano con excelencia y virtuosismo. Cada día él hace unas caminatas cortas al aire fresco, excepto en el invierno cuando la nieve o el hielo lo impide. Nosotros siempre lo exhortamos a que camine más y que fume menos, pero él siempre responde “Aceite de plátano”. Al abuelo le gusta ser moderno en su lenguaje.* It is a short reading passage that has evolved into a ubiquitous metric of reading ability and speech intelligibility.

### 2.4. Prosodic Analysis

To obtain the prosodic features of speech recordings by means of automatic prosodic transcription, a novel algorithm to automatically detect syllable nuclei was used. The proposed algorithm is mainly based on the method described in [8] for speech rate detection.

**Figure 2:**
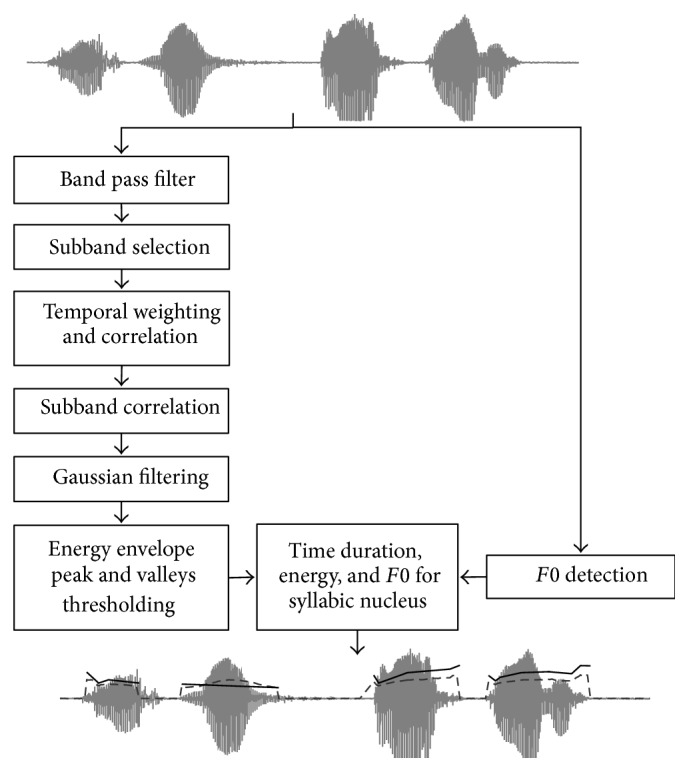
Algorithm block diagram for features set (adapted from [8]).

The overall procedure is illustrated in Figure 2. The input speech signal is processed in parallel to obtain an automatic estimation of both syllable nuclei and fundamental frequency (F0 detection). In order to increase the temporal resolution of the energy envelope, the downsampling process is removed; also smaller windows size (10 ms) and overlap (5 ms) are used in the temporal weighting stage. Then, the syllabic nuclei are detected using the same threshold mechanism described in the peak counting stage in [8].

The vowel nucleus is the place where the pitch estimation reaches a local maximum and this phenomenon is relative to the syllable boundaries because simultaneous changes of intensity, spectral energy distribution, and voicing partially hide the perception of the pitch changes [9]. This feature is more evident for stop and fricative consonants and less significant for liquids and nasals [10]. Consequently the edges of the syllabic nucleus are more suitable for the detection of noticeable changes in the fundamental frequency than the syllable border. The boundaries of the syllable nucleus are estimated by the nearest minimum related to the detected peak on the energy envelope or by vocal activity limits, provided by the robust algorithm for pitch tracking (RAPT) [11].

For each syllable nucleus obtained, a number of features related to measures of intensity, duration, and fundamental frequency are estimated (see central bottom block in Figure 2). Duration and fundamental frequency features are given in milliseconds (ms) and semitones (ST), respectively. Expressing fundamental frequency in semitones diminishes gender differences as suggested in [12, 13].

For our research twelve prosodic features were calculated based on the syllable nucleus position obtained by the novel prosodic method as follows:

1. speech time (SPT): total speech time from first syllable to last syllable produced,
2. number of pauses (NPU): total number of silences; a gap between two consecutive syllables over 0.3 s that was considered silence,
3. proportion of pause (PPU): total number of pauses over 0.3 s divided by the total amount of time spent speaking expressed in seconds,
4. phonation time (PHT): total time of all syllables produced plus silences lower than 0.3 s,
5. proportion of phonation (PPH): total time of all syllables produced plus silences lower than 0.3 s divided by the amount of time,
6. speech rate (SPR): total number of syllables produced in a given speech sample divided by the amount of total time (including pause time),
7. articulation rate (ARR): total number of syllables produced in a given speech sample divided by the amount of time taken to produce them in seconds,
8. number of syllables (NSY): total number of syllables produced along the speech sample,
9. mean of syllables duration (MSD): mean time of all syllables in seconds,
10. standard deviation of F0 (SDF): standard deviation of fundamental frequency of all syllables produced along speech sample,
11. maximum variation of F0 (MVF): maximum difference between higher and lower values of fundamental frequency along speech sample,
12. mean of F0 (MFF): mean of fundamental frequency of all syllables produced.

### 2.5. Automatic Classification

Many classification techniques have been developed with remarkable performance in the last decades [14]. Since the main goal of this research is to find a set of prosodic parameter with discriminative potential for identifying mild dementia a well-known classifier was selected. In [15] we explored the use of Random Forest for a similar task, but now we have found that better results can be achieved using the Support Vector Machine (SVM) classification technique. Therefore our automatic classification of reading speech is based on SVM technique to evaluate how well the proposed features predicted participant's group membership. The results are evaluated in terms of accuracy (*Accu*), sensitivity (*Sens*), and specificity (*Spec*) measurements [16]. A cross validation technique was used to avoid overfitting, that is, a discriminant function to be created with the same data used later for testing. Specifically the leave-one-out method of cross validation was applied. It involves generating the discriminant function on all but one of the participants (*n* − 1) and then testing for group membership on that sample. The process is repeated for each sample (*n* times) and the percentage of correct classifications is generated through averaging for the *n* trials. In our case *n* is equal to the total number of participants (*n* = 20), and, in each iteration of the cross validation method, one fold is used for testing and the other nineteen folds are used for training the SVM classifier.

## 3. Results

The goal of our experiments was to evaluate the potential of selected features for automatic measurement of the impairment cognition through prosodic analysis.  Table 2 contains descriptive statistics for these measures showing the mean, standard deviation and range for every prosodic measure on both groups (MD and non-MD).

Visual inspection reflects that probable differences between subjects with mild dementia and healthy subjects could be found on some measures like SPT, NPU, NSY, SDF, and MFF. Other measures like PPU, PPH, ARR, and MSD show no significant differences suggesting that these measures have no power to discriminate between classes.

Hence, we performed a Kolmogorov-Smirnov test (KS-test) to determine which set of features can significantly discriminate between the two groups (MD and non-MD) [17]. The KS-test has the advantage of making no assumption about the distribution of data. This nonparametric test for the equality of continuous, one-dimensional probability distributions can be used to compare a sample with a reference probability distribution (one-sample KS-test) or to compare two samples (two-sample KS-test). The null distribution of this statistic is calculated under the null hypothesis that the samples are drawn from the same distribution (in the two-sample case). The two-sample test is one of the most useful and general nonparametric methods for comparing two samples, as it is sensitive to differences in both location and shape of the empirical cumulative distribution functions of the two samples [18]. Nevertheless with a KS-test, we cannot guarantee finding the best set of features to reach the maximum performance of the SVM classifier but we believe it can provide an acceptable first approximation to it. Statistical analyses were conducted using IBM SPSS v21 [19], and the desired significance level of 0.05 was used.

Summary of results of the hypothesis (*h*), the *p* values for KS-test, and a ranking of level of significance for each feature based on the lower *p* values are shown in Table 3. Despite the small sample used, the ranking attempt illustrates the relative importance of these variables for discriminating between healthy and mild dementia.

Using this information three sets of features (presented in Table 4) were defined according to three different levels of discrimination. The first set included those features with significant differences (*p* < 0.05) between both groups (SDF and MFF). The second set contained features with slight differences (*p* < 0.5) between MD and non-MD participants, for example, PTH, SPR, NSY, or MVF. Features with no significant differences (*p* > 0.5) between groups: SPT, NPU, PPU, PPH, ARR, and MSD were included in the third set. We think that the small sample size may have resulted in this lack of significance and that these temporal measures may yet offer additional explanatory power.

SVM was carried out to determine how well the proposed groups of level of significance predicted participants' group membership. Classification results were obtained using different combinations of the prosodic features included in the three significance groups summarized in Table 4.

As it can be seen in Table 5, using this classification strategy, the best accuracy of 75.0% was achieved using only the set of features in the significant group. While interpreting the results of Table 5, we should note that the classification algorithm did not take advantage from increasing the number of features. Even so these results could be considered relatively good based on the size of the data and published results elsewhere [20–22] as depicted in Table 6.

The former method of feature selection is one of the statistical methods frequently used in similar studies to evaluate the level of significance for measurement under analysis [23, 24]. Due to the nature of this method (KS-test over individual features) it cannot guarantee maximum accuracy for a classifier able to model complementary information between features. The set of features that could represent in a better way both classes (MD and non-MD) cannot be determined by the level of significance of individual features. Even the combination of different features can either improve or worsen the final classification. Consequently one critical question arises and still needs to be answered: how to choose the set of features to able to reach the maximum classification rate for a given classification technique. Trying to answer this question, in the next step, we proceeded with the evaluation of the SVM using all the possible combination of the features obtained from the prosodic analysis. In our work a total of 4094 combinations from 12 features without repetition were tested to find the best feature set. The best classification rates, using leave-one-out cross validation, for each combination of a features amount are shown in Table 7.

Results in Table 6 indicate that the relation between features and the way each affects others in the pattern classification is not well known. The highest accuracy value was obtained for the combination of four features, including the best (SDF) and worse (MSD) ranked in Table 3. We also should note that increasing the number of features does not guarantee an increment in the classification rate.

## 4. Conclusions

The main goal of this pilot study was to investigate the potential use of automatically extracted prosodic features in the diagnosis of mild dementia in elderly adults. The results demonstrate the existence of significant measureable prosodic differences between the performance of healthy participants and patients with mild dementia in reading speech. Features like ARM, MSD, SDF, and MFF were identified as having a higher discriminative power. Furthermore, due to the relative simple and low cost methodology, the technique for the screening of moderate cognitive impairment is easy to spread out. This study lays the foundation for future research using a larger number of participants and other speech features either in time or in spectral and cepstral domain. In this way, definitive conclusions of prosodic analysis to identify mild dementia could be drawn.

## Figures and Tables

**Table 1:** Participant demographic data.

Item	Group	
	MD	Non-MD
Number of patients	10	10
Male	6	9
Female	4	1
Average age (years)	80.3	78.9
Average years of education (years)	4.9	7.8

**Table 2:** Prosodic features for mild dementia patients (MD) and healthy controls (non-MD).

Features	MD		Non-MD	
	Mean (SD)	Range	Mean (SD)	Range
SPT	156.4 (56.4)	75.0–234.2	127.9 (64.6)	59.2–249.7
NPU	81.3 (38.2)	32–132.0	62.1 (33.7)	27.0–124.0
PPU	54.7 (16.6)	30.1–78.2	52.2 (10.8)	30.7–64.8
PHT	63.2 (13.4)	50.3–90.1	55.3 (16.9)	33.5–107.2
PPH	45.2 (16.6)	21.7–69.8	47.7 (10.8)	35.1–69.2
SPR	2.1 (0.8)	1.2–3.8	2.3 (0.5)	1.6–3.6
ARR	4.8 (0.7)	3.6–5.9	4.8 (0.6)	4.3–6.3
NSY	306.6 (67.6)	225–449	266.8 (75.1)	133–401
MSD	0.1 (0.0)	0.0–0.1	0.1 (0.0)	0.0–0.1
SDF	42.0 (13.5)	24.5–68.6	29.3 (8.7)	17.6–46.6
MVF	279.3 (80.2)	143–377	232.9 (109.2)	99–375
MFF	174.4 (40.6)	108.0–219.9	138.2 (27.7)	105.9–192.3

**Table 3:** Statistic analysis results.

Features	Kolmogorov-Smirnov		
	*h*	*p* values	Ranking
SPT	0	0,675	7
NPU	0	0,675	8
PPU	0	0,675	9
PHT	0	0,312	3
PPH	0	0,675	10
SPR	0	0,312	4
ARR	0	0,675	11
NSY	0	0,312	5
MSD	0	0,974	12
SDF	1	0,030	1
MVF	0	0,312	6
MFF	1	0,032	2

**Table 4:** Level of significance based on *p* values for prosodic features.

Significant (SIG)	Possibly significant (PSIG)	Nonsignificant (NSIG)
SDF	PTH	SPT
MFF	SPR	NPU
	NSY	PPU
	MVF	PPH
		ARR
		MSD

**Table 5:** SVM classification results for significant group combination.

Group	Accu	Sens	Spec
SIG	**75.0**	**72.7**	**77.7**
PSIG	35.0	36.3	33.3
NSIG	45.0	46.1	42.8
SIG-PSIG	60.0	58.3	62.5
SIG-NSIG	65.0	66.7	63.6
PSIG-NSIG	30.0	33.3	25.0
SIG-PSIG-NSIG	65.0	61.5	71.4

**Table 6:** Summary of dataset size and classification accuracies reported in previous works.

Previous works	Participants (patients)	Classification accuracies
Lehr et al. [20]	72 (35)	75.4%–81.5%
Thomas et al. [21]	85 (50)	58.8%–75.3%
Bucks et al. [22]	24 (8)	87.5%

**Table 7:** SVM classification results for best features combination.

Features	Accu	Sens	Spec
SDF	75.0	69.2	85.7
PHT-MFF	75.0	72.7	77.7
NPU-PHT-MFF	80.0	80.0	80.0
ARR-MSD-SDF-MFF	**85.0**	**81.8**	**88.8**
NPU-NSY-SDF-MVF-MFF	80.0	80.0	80.0
NPU-PPH-NSY-SDF-MVF-MFF	80.0	80.0	80.0
NPU-PPU-PPH-NSY-SDF-MVF-MFF	80.0	80.0	80.0
SPT-NPU-PPU-PPH-NSY-SDF-MVF-MFF	80.0	80.0	80.0
SPT-NPU-PPU-PHT-PPH-SPR-ARR-NSY-MFF	75.0	72.7	77.7
NPU-PPU-PHT-PPH-ARR-NSY-MSD-SDF-MVF-MFF	70.0	66.6	75.0
SPT-NPU-PPU-PHT-PPH-SPR-ARR-NSY-MSD-SDF-MFF	70.0	66.6	75.0
SPT-NPU-PPU-PHT-PPH-SPR-ARR-NSY-MSD-SDF-MVF-MFF	65.0	61.5	71.4

## References

[1] Llibre J. J. (2012). Aging and dementia: implications for the scientist community, public health and Cuban society. *Revista Anales de la Academia de Ciencias de Cuba*.

[2] Folstein M. F., Folstein S. E. (1975). ‘Mini-mental state’. A practical method for grading the cognitive state of patients for the clinician. *Journal of Psychiatric Research*.

[3] Morris J. C. (1993). The clinical dementia rating (CDR): current version and scoring rules. *Journal of Neurology*.

[4] Buschke H., Kuslansky G., Katz M. (1999). Screening for dementia with the Memory Impairment Screen. *Neurology*.

[5] Deramecourt V., Lebert F., Debachy B. (2010). Prediction of pathology in primary progressive language and speech disorders. *Neurology*.

[6] Mesulam M., Wicklund A., Johnson N. (2008). Alzheimer and frontotemporal pathology in subsets of primary progressive aphasia. *Annals of Neurology*.

[7] Darley F. L., Aronson A. E., Brown J. R. (1975). *Motor Speech Disorders*.

[8] Wang D., Narayanan S. S. (2007). Robust speech rate estimation for spontaneous speech. *IEEE Transactions on Audio, Speech and Language Processing*.

[9] House D. The influence of silence on perceiving the preceding tonal contour.

[10] Mertens P. The prosogram: semi-automatic transcription of prosody based on a tonal perception model.

[11] Talkin D., Kleijn W. B., Paliwal K. K. (1995). A robust algorithm for pitch tracking (RAPT). *Speech Coding and Synthesis*.

[12] t’Hart J. (1976). Psychoacoustic backgrounds of pitch contour stylization. *IPO Annual Progress Report*.

[13] t'Hart J. (1981). Differential sensitivity to pitch distance, particularly in speech. *The Journal of the Acoustical Society of America*.

[14] Duda R. O., Hart P. E., Stork D. G. (2001). *Pattern Classification*.

[15] Espinoza-Cuadros F., Garcia-Zamora M. A., Torres-Boza D. (2014). A spoken language database for research on moderate cognitive impairment: design and preliminary analysis. *Advances in Speech and Language Technologies for Iberian Languages*.

[16] Metz C. E. (1978). Basic principles of ROC analysis. *Seminars in Nuclear Medicine*.

[17] Massey F. J. (1951). The Kolmogorov-Smirnov test for goodness of fit. *Journal of the American Statistical Association*.

[18] Schultz L. M. (2010). P. Sprent & N.C. Smeeton (2007). Applied nonparametric statistical methods (4th ed.). *Psychometrika*.

[19] Norušis M. J. (2004). *SPSS 13.0 Advanced Statistical Procedures Companion*.

[20] Lehr M., Prudhommeaux E., Shafran I., Roark B. Fully automated neuropsychological assessment for detecting mild cognitive impairment.

[21] Thomas C., Kešelj V., Cercone N., Rockwood K., Asp E. Automatic detection and rating of dementia of Alzheimer type through lexical analysis of spontaneous speech.

[22] Bucks R. S., Singh S., Cuerden J. M., Wilcock G. K. (2000). Analysis of spontaneous, conversational speech in dementia of Alzheimer type: evaluation of an objective technique for analysing lexical performance. *Aphasiology*.

[23] Pakhomov S. V. S., Marino S. E., Birnbaum A. K. (2013). Quantification of speech disfluency as a marker of medication-induced cognitive impairment: an application of computerized speech analysis in neuropharmacology. *Computer Speech & Language*.

[24] Gonzalez-Moreira E., Torres-Boza D., Garcia-Zamora M. A., Ferrer C. A., Hernandez-Gomez L. A. (2015). Prosodic speech analysis to identify mild cognitive impairment. *VI Latin American Congress on Biomedical Engineering CLAIB 2014, Paraná, Argentina 29, 30 & 31 October 2014*.

